# Telehealth in the Context of COVID-19: Changing Perspectives in Australia, the United Kingdom, and the United States

**DOI:** 10.2196/19264

**Published:** 2020-06-09

**Authors:** Malcolm Fisk, Anne Livingstone, Sabrina Winona Pit

**Affiliations:** 1 De Montfort University Leicester United Kingdom; 2 Global Community Resourcing Brisbane Australia; 3 University Centre for Rural Health School of Medicine University of Western Sydney Lismore Australia; 4 University Centre for Rural Health School of Medicine and Health Sciences University of Sydney Lismore Australia

**Keywords:** telehealth, COVID-19, SARS-CoV-2, public health, older people, resource allocation, aged care, innovation, pandemic, telemedicine

## Abstract

**Background:**

On March 12, 2020, the World Health Organization declared the coronavirus disease (COVID-19) outbreak a pandemic. On that date, there were 134,576 reported cases and 4981 deaths worldwide. By March 26, 2020, just 2 weeks later, reported cases had increased four-fold to 531,865, and deaths increased five-fold to 24,073. Older people are both major users of telehealth services and are more likely to die as a result of COVID-19.

**Objective:**

This paper examines the extent that Australia, the United Kingdom, and the United States, during the 2 weeks following the pandemic announcement, sought to promote telehealth as a tool that could help identify COVID-19 among older people who may live alone, be frail, or be self-isolating, and give support to or facilitate the treatment of people who are or may be infected.

**Methods:**

This paper reports, for the 2-week period previously mentioned and immediately prior, on activities and initiatives in the three countries taken by governments or their agencies (at national or state levels) together with publications or guidance issued by professional, trade, and charitable bodies. Different sources of information are drawn upon that point to the perceived likely benefits of telehealth in fighting the pandemic. It is not the purpose of this paper to draw together or analyze information that reflects growing knowledge about COVID-19, except where telehealth is seen as a component.

**Results:**

The picture that emerges for the three countries, based on the sources identified, shows a number of differences. These differences center on the nature of their health services, the extent of attention given to older people (and the circumstances that can relate to them), the different geographies (notably concerned with rurality), and the changes to funding frameworks that could impact these. Common to all three countries is the value attributed to maintaining quality safeguards in the wider context of their health services but where such services are noted as sometimes having precluded significant telehealth use.

**Conclusions:**

The COVID-19 pandemic is forcing changes and may help to establish telehealth more firmly in its aftermath. Some of the changes may not be long-lasting. However, the momentum is such that telehealth will almost certainly find a stronger place within health service frameworks for each of the three countries and is likely to have increased acceptance among both patients and health care providers.

## Introduction

### Context

In March 2020, the nature and virulence of the coronavirus disease (COVID-19) became a matter of urgent debate. This brought telehealth into focus as a potential tool to help provide services without the need for direct face-to-face contact. Older people, as major users of telehealth services and the age group most likely to die as a result of a COVID-19 infection, were positioned to become beneficiaries of any expanded range of these services. This may especially be the case since older people in isolation may be at risk of depression and anxiety [1,2].

The focus of this paper is on the 2-week period beginning March 12, 2020, when the World Health Organization announced that the COVID-19 outbreak was a pandemic. Notably, on this date, there was high or accelerating numbers of cases and related deaths in China (where the disease was first reported), South Korea, Iran, Italy, and Spain. Worldwide there were 134,576 reported cases and 4981 deaths. By March 26, just 2 weeks later, there were 397,289 reported cases and 24,073 deaths, both reflecting increases of over 300% [3]. These global increases were reflected in increases for each of the three countries (see Table 1).

Older people have the highest risk of mortality from COVID-19. The risk may vary per country or per region depending on various factors such as screening strategies and population distributions. The Chinese Center for Disease Control and Prevention reported that, from 72,314 cases, there was a case-fatality rate (CFR) of 8.0% for those aged 70-79 years and a 14.8% CFR for those 80 years or older [4]. The highest CFR was for people with cardiovascular disease, diabetes, chronic respiratory disease, hypertension, and cancer. Men had a higher risk of death than women (2.8% vs 1.7%). Italy has shown higher CFRs for people aged 70-79 years (19.1%) when compared to China (8.0%) [5]. Gender differences for cases and deaths were indicated for Italy and Germany, where men were, according to initial statistics, over 30% more likely to have the disease or die from it [6,7]. Such differences were noted as coming with provisos that relate to, for example, lifestyles and the extent of people’s employment in relevant caring tasks [8,9]; overall (ie, for all projections at this early stage of the disease), there is further statistical uncertainty in view of data relating solely to “confirmed” cases and the omission of an “unknown” number of people who are asymptomatic [10].

**Table 1:** Coronavirus disease cases and deaths worldwide in Australia, the United Kingdom, and the United States from March 12 to 16, 2020.

Country	March 12, 2020		March 26, 2020		Increase	
	Cases, n	Deaths, n	Cases, n	Deaths, n	Cases, n (%)	Deaths, n (%)
Australia	156	3	3050	13	2894 (1955)	10 (433)
United Kingdom	590	10	11,658	578	11,068 (1976)	568 (5780)
United States	1697	41	85,435	1295	83,737 (5034)	1254 (3159)
World	134,576	4981	531,865	24,073	397,289 (395)	19,092 (483)

### Definition of Telehealth

A note on the definition of telehealth is necessary in view of its uneasy position in relation to telemedicine, telecare, technology-enabled health, and digital health—terms that may overlap and are sometimes used interchangeably. Mobile health (mHealth; and the use of apps) are also included. Differences in understanding are indicated in the country profiles below.

The definition of telehealth offered here is encompassed by the term digital health. Its origin lies in the European Code of Practice for Telehealth Services [11]. Telehealth, it affirms, is “the means by which technologies and related services concerned with health and well-being are accessed by people or provided for them irrespective of location.” This definition fits with Wootton’s [12] description of telemedicine as “health care carried out at a distance,” with both reflecting a person- (or patient-) centered, as opposed to a technology-driven, approach. Either can be viewed as suitable to underpin potentially new norms for health service provision, in part forced by the COVID-19 pandemic.

### Benefits and Barriers of Telehealth

The reported benefits of telehealth have focused on cost, choice, and convenience. In respect to cost, much debate has taken place on the extent of financial savings that might be realized. The most substantial study of telehealth interventions, that of the “Whole System Demonstrators” (WSDs), started in 2010 and involved 3000 patients in England. It was reported that, although there was a reduction in hospital admissions, telehealth did not “seem to be a cost-effective addition to standard support and treatment” [11,13]. An ensuing study in Northern Ireland, with just under 4000 patients, noted “no evidence within the dataset of any marked impact of telehealth services on hospitalisations and hospital-based service usage” [14]. Both studies related to interventions involving the use of devices such as vital sign monitors linked to home hubs characteristic of “telecare” services that operate throughout the United Kingdom.

In contrast, an Australian study in 2013-2014 that involved nearly 300 telehealth patients explored the benefits of both vital signs monitors and technologies that included participant videoconferencing capabilities and messaging features. It, like the WSDs in the United Kingdom, found a reduction in hospital admissions but, importantly, also found a significant improvement in participants’ health literacy and health behaviors, together with reported improvements in anxiety, depression, and quality of life [15,16]. The Australian study, by taking a more person-focused approach, at least *touched* on some of the potential benefits of telehealth that are not concerned with cost-effectiveness (as seen by provider organizations and funding bodies).

Other small-scale studies have taken this further, emphasizing the convenience of such services, especially when accessed by people in rural areas and, therefore, obviating the need to travel [17]. Although personal contact in the form of *presence* and *touch* is beyond telehealth, at least there is some help toward addressing the twin scourges of loneliness and social isolation that can afflict some older people. Linked with this is a real ability to make connections that give comfort and companionship to (older) people when they are dying, access to interpreters and signers where necessary, and even provide cognitive behavioral therapy.

Prominent among telehealth technologies are health apps, with their ubiquity increasing in the context of smartphone use, including messaging for mindfulness-based therapy for patients with cancer [18] or providing “digital therapy” for people with mental health needs [2]. There is an immense range of health-related apps, totaling over 300,000 in iTunes and Google Play in 2017, though the quality of most apps was considered as leaving much to be desired [19].

Known barriers to uptake of telehealth, aside from cost-effectiveness, center around the lack of (suitable) information technology and the security of communication links regarding personal (including health) data [2,20]. In addition, there are concerns regarding the impact on patient rapport, workforce, liability and legal issues, and time constraints [21]. Other barriers include the need to rethink business models and to overcome financial barriers including incentives, billing, and both initial and longer-term funding. Finally, some are concerned that telehealth is of lower efficacy; this clearly being the case where *face-to-face* contact with patients is necessary, for example, to undertake clinical examinations [17]. It is also of concern if there would be any deferring (by older people in the context of COVID-19) of making contact with health services that could assist in relation to, for example, pain, weight loss, and diminution of strength, or if any circumstances were overlooked that could relate to, for example, isolation and its consequences (eg, lack of food intake, depression, and even suicidal thoughts). Additionally, the COVID-19 outbreak could lead to a “mental health spike” [2].

Finally, there are the twin technological barriers that relate to the limitations or unreliability of internet connections (a matter that relates, in part, to the geographies of each country but also to the capacity and, therefore, the efficacy of remote connections) and the interoperability of the technologies concerned.

In relation to the positive aspects of telehealth, the need for future research has been called for by Dinesen et al [22] who affirmed the need to “identify...factors that *promote* [our emphasis] telehealth acceptance, such as human-technology interaction, organization of the health care system and social factors.”

### COVID-19 and Telehealth

The COVID-19 pandemic casts telehealth in a new light because it is accessed by people directly from their homes. The use of telehealth “may reduce the likelihood of viral transmission by limiting person-to-person contact, while enabling people with the virus to be treated for viral symptoms and their normal medical conditions” [23]. Health professionals meanwhile (as is already the case for extant telehealth services) are able to undertake more work from home. In addition, telehealth can be seen as safeguarding both health and social care workers, and (older) people who access such services *at least for those tasks that do not require physical contact*.

Hollander and Carr [24] pointed to the merits of telehealth enabling the diversion of people, where appropriate, from centers that deal with “emergencies” to “nurse triage lines” or for scheduled video consultations. They affirmed that remote screening and diagnosis (or referrals) can potentially reduce exposure for health care workers and other patients, as it will allow patients “to bypass the ED (Emergency Department) and be placed directly in a hospital bed.” Technologies (whether via tele- or video consultations) can, they argued, inform and motivate people in relation to their lifestyles or the (self-) management of different conditions.

We can envisage, furthermore, the increased use of self-test kits with these and other vital sign measurement devices (eg, for blood pressure and respiratory function), contributing to a devolution of more traditional health care to the home. At least some of the matters that tie us to “the delivery of services in bricks-and-mortar campuses and clinics,” where infection transmission is too easily facilitated, could be loosened and even make such institutions “largely unusable” [25].

It is only a small additional step to recognize the potential of telehealth through the use of artificial intelligence (AI) with, for example, remote screening via video that can recognize and record voice, facial expressions, attention, skin pallor, movement, and other signs for assessment and diagnostic purposes. However, published work in English on the use or potential use of AI in the context of both telehealth and COVID-19 is currently absent except for identifying disease outbreaks and learning from patterns of spread [26].

Particular barriers in relation to telehealth development in response to COVID-19 for three countries are noted in the Results section. Some are being addressed by changes to legislation and regulation, finance, and support programs. Protocols to guide at least video consultations were either already in place or under review. Most health and social care professionals, faced with the new demands of COVID-19, are therefore on a steep learning curve relating to the virus itself and necessary operational changes.

This paper examines the extent to which Australia, the United Kingdom (consisting of England, Scotland, Wales, and Northern Ireland), and the United States have, as a consequence of the pandemic, implemented telehealth as a tool to help older people who may live alone, are frail, or are self-isolating; identify those who may have a COVID-19 infection; and give support and facilitate treatment where necessary.

## Methods

Three countries were explored: Australia, the United Kingdom, and the United States. The focus is on the 2-week period starting on March 12, 2020, when the World Health Organization announced the COVID-19 pandemic. This paper reports on the activities and initiatives taken by governments or their agencies (at national or state levels) in the three countries and the publications or guidance issued by professional, trade, and charitable bodies. Different sources of information are drawn upon that point to the perceived likely benefits of telehealth in fighting the pandemic.

Accessing the relevant sources of information for each of the countries was facilitated in large part by the knowledge and networks of the coauthors. It involved internet searches, scrutiny of media reports, and the use of contacts who work in practice or are involved in consultancy (including those who are acknowledged at the end of this paper). The focus of attention was on national (or federal) policy initiatives that would impact, through changes in funding frameworks or other strategic measures, on practice within regional, state, or territory jurisdictions.

During the period of study, the coauthors explored the burgeoning range of publications online (in the form of newsletters and blogs), some of which bore testimony to the escalation in use of tele- and video consultations, and reactions (mainly of service providers) to the funding and policy changes that were being enacted or signaled. In the wake of these a smaller but important number of academic articles were accessed—these, in general, revisited earlier work and began to re-evaluate the potential of telehealth in the new context.

The different governmental structures of the three countries, and the fragmented nature of telehealth services therein, means that they cannot be taken as representative of any wider range of countries. This must be a matter for future study.

## Results

### Australia

On March 11, 2020, the Australian government announced an AUS $2.4 billion (US $1.6 billion) health package to combat COVID-19. AUS $100 million (US $68 million) was promised to fund a “new Medicare service,” at no cost for patients, concerned with telehealth consultations via phone or video (eg, Skype) by general practitioners (GPs), specialists, nurses, and mental health allied health workers. The service would be available for COVID-19–related consultations and, more widely, to people at greater risk of COVID-19 infection, including those older than 70 years (or older than 50 years for Aboriginal or Torres Strait Islanders), people with chronic conditions or who are immunosuppressed, women who are pregnant, and parents with new babies. A free 24/7 national triage phone line was also to benefit from additional funding of AUS $50.7 million (US $34.4 million). In addition, AUS $25 million (US $17 million) was earmarked to fund Australians in isolation and at-risk groups to file their medication prescriptions online and have medicines home delivered free-of-charge [27].

This urgent initiative followed a call from the Royal Australian College of General Practitioners (RACGP) on March 6, 2020, for the government to “relax current restrictions around telehealth services by removing geographical constraints and permitting GPs to interact with their patients irrespective of location” [23]. Other bodies such as the Australian Medical Association, Australian College of Rural and Remote Medicine (ACRRM), and the Rural Doctors Association of Australia added their voice. Snoswell et al [23] noted that previous government telehealth funding had been made in response to droughts and bushfires, and on March 23, the Australian Government allowed vulnerable health care professionals who were authorized to use telehealth “item numbers” (ie, reimbursable) for all consultations with all their patients [28].

Australia already had relatively well-established telehealth services with some 150,000 “visits” having taken place from “rural and remote communities” in 2018 [23]. This is despite that fact that medical students are not exposed much to telehealth in their training, despite a realization of its potential benefits. In a 2018 study, they expressed preference for face-to-face consultations [17].

The ACRRM had published a standards framework (in 2016) that promoted the use of telehealth services for remote communities [29]. Guidelines for operating videoconference calls were already in place, such as from the Royal Australasian College of Physicians [30]; although, these required a GP, practice nurse, or Aboriginal health worker to be present with the patient during consultations. There remain barriers, however, to service operation in view of people having to have “access to a videoconferencing platform and internet connection” despite, as noted by Snoswell et al [23], that these could be freely or cheaply done via a “tablet or PC”. The broader context for Australia had been set in the nation’s digital health strategy that called for “widening access to telehealth services” [31].

Finally, on March 30, 2020, the Australian government announced funding of AUS $669 million (US $454 million) for the rollout of a universal telehealth model for all Australians to enable health care access through tele- or video consultations from home until September 30, 2020 [32]. There were multiple benefits: the new model enabling a reduction in COVID-19 exposure for both patients and health care providers, a maintaining of the primary health care frontline, and a reduction in the demand for personal protective equipment and emergency departments. It also helped people to stay at home, therefore, supporting compliance with self-isolation and quarantine requirements. Providers were expected to adopt either a 100% remote business model or a hybrid model of service provision. Face-to-face consultations were recognized as still being needed where physical examination was required or where technology could not be used (eg, for a confused patient without support). Practices would need to create new workflows, and some local primary health networks were guided in these tasks [33].

In addition, on March 30, 2020, further funding (AUS $74 million [US $50 million]) was provided to support telehealth consultations for those with mental health needs, including the development of a digital mental health portal and a “coronavirus hotline” for well-being and online support for health workers. Specifically for older people, AUS $10 million (US $6.8 million) was assigned to the existing community visitors scheme (and to train volunteer visitors) to combat social isolation caused by COVID-19–imposed visiting restrictions. Such volunteer visitors will connect with older people both online and by phone.

Elsewhere, the growth in the use of apps was noted by Scott et al [19], who recognized their usefulness in relation to certain health conditions. They set out a comprehensive framework by which their merits could be assessed. An “App Evaluation Model” for mental health apps is available from the American Psychiatric Association [34]. Apps were already being trialed within some local primary health networks in Australia [35] and in the United Kingdom [36]. By March 27, 2020, a European Commission funded project had pointed to 19 mHealth “solutions” to help with the COVID-19 outbreak [37].

Given the role of apps as a means that people can self-manage and at the same time share information (eg, on heart rate and respiration) with health services, their *potential* importance as a tool of telehealth in the context of the COVID-19 pandemic is clear. Research has demonstrated that, if health care providers discuss the use of health apps with their patients, they are generally willing to use them to manage their chronic conditions [38], but such use in Australia is still in its infancy. COVID-19 might accelerate their uptake and use.

Furthermore, well-developed “aged care” services are important throughout Australia. These include the usual range of services in care homes and the wider community (home care), and have often been underpinned by “social alarms” (personal response systems) or telecare. In this sector, although not clinician-led, there is a rapidly growing awareness of telehealth’s potential in supporting older people at home and of the tools that are available to help with this. The Aged Care Industry IT Council (ACIITC; that draws together Aged Care Services Australia and Leading Age Services Australia) mapped and documented recent technology changes [16]. This helped multiple aged care services to re-evaluate their roles in the context of such technologies and, where they had not already done so, to look at service cultures, operational procedures, and related training.

A leading Australian example of an *aged care* service that crosses over from the social to health care sectors is provided by Feros Care. This *aged care* service provider has partnered, for its home care services, with Google to facilitate older people’s use of Google Assist, thus, giving them (and caregivers) access to the organization’s portal and a widening range of information and other services. Information gathered regarding service use is envisaged as a prelude to using AI to monitor well-being [16].

The range of technologies documented in the work of the ACIITC was substantial and carries importance in the COVID-19 context. It ranged from apps and voice assistants (including Google Assist, Amazon’s Alexa, and Apple’s Siri) to fall-detection devices and socially assistive robots—with consideration also being given to related developments around smart homes and the internet of things. Importantly the report recognized “touch points” with clinicians. This is because of the extent that such technologies are now able to provide lifestyle and physiological data that can both help people to remain in good health and safely manage any health conditions, a matter that carries greater importance when self-isolating. It is only a small step thereafter to consider (as previously noted) how AI can be used [39] to facilitate not just monitoring (with necessary safeguards around privacy) but also diagnosis and treatment.

In the meantime, levels of awareness of the role of telehealth in relation to COVID-19 are rapidly rising. Helping this was the Digital Health Cooperative Research Centre webinar on March 18, 2020, titled “COVID-19 and Digital Technology: the Roles, Relevance and Risks of Using Telehealth in a Crisis” [40].

The resources, guidelines, training, online forums, and directory of telehealth care specialist and generalists maintained by the ACRRM may, in this context, assist health care organizations in setting up, reshaping their services, and supporting their workforces through the transitions [41]. The RACGP, furthermore, was poised to release a checklist on how to set up good clinical care in the age of telemedicine. More broadly, the Australian Digital Health Agency, having consulted on the issue of interoperability in 2019, aimed to publish a “National Health Interoperability Roadmap” [42].

However, as for all three countries, the *reach* of new telehealth initiatives to older people in Australia, despite the urgency around COVID-19, is uncertain. Many people, disproportionately those with the greatest needs, may not have (or cannot afford) smartphones or computers. Some, depending on location, have poor (or no) connectivity—albeit alleviated by the fact that many can use a landline to consult with their health care provider. Others, maybe many, may find it hard to consult over the phone and could forgo their health care *visits* until the pandemic is finished, with this potentially leading to other health care complications, the implications of which have not yet been adequately considered.

In summary, there is a good range of operational telehealth services in Australia (that offer tele- and video consultations), notably in rural areas, and such services may be able to further develop their wares as a consequence of the government’s investment promise. Furthermore, the existing range of aged care services and the extent of their recognition in the role of new technologies is significant. This makes Australia relatively well positioned to respond to the COVID-19 challenge and to develop telehealth services in ways that respond to both health and social care needs.

### United Kingdom

On March 12, 2020, the United Kingdom was moving to a “containment” phase in its response to the COVID-19 pandemic. The Prime Minister affirmed that, in this phase, “many more families will lose loved ones before their time” [43]. Telehealth did not have a place in the UK government’s plans at this point; although Scotland had announced on March 10 that they were “accelerating” an investment of £1.24 million (US $1.5 million) plus £8 million (US $10 million) “implementation” costs to support video consultations, already used in rural areas, more widely, including for GP consultations [44].

Impetus for the UK action was added to through a publication from the Imperial College London. On March 16, 2020, Ferguson et al [45] modeled the potential of nonpharmaceutical interventions for the United Kingdom and the United States, aimed at reducing contact rates and disease transmission. It pointed to the possibility that 81% of both populations would catch the disease if control measures were not put in place. The control measures included in the modeling were “case isolation in the home,” “voluntary home quarantine” of *all* household members, and “social distancing.” These, of course, have a severe impact on older people.

On March 17, 2020, NHS England issued a notice to health trusts, health service commissioners (procurers), and providers, including GP services. This called for the agencies in question to “support the provision of telephone-based or digital- and video-based consultations, and advice for outpatients,” and for general patient consultations to be undertaken by GPs and other health care staff. For the latter, the “roll out” of such practices would, it was considered, be accompanied by increased use of email and text messaging. “Face to face appointments,” the notice stated, “should only take place when absolutely necessary.”

The limited promotion of telehealth on a UK-wide basis in response to COVID-19 is likely because of the general lack of developed services (the exception being Scotland). This is despite what has been recognized as a sizeable market for such services—a major part of which was relating to mHealth and the use in the United Kingdom of apps and smart phones [46]. There are, however, many established social alarm (personal response systems) and telecare services. A leading UK example (in the north of England) of a telehealth service that crosses over from the health care to the social care sector is that provided by the Airedale NHS Foundation Trust. The “hub” provides varied services including telemonitoring, tele-coaching, the provision of advice, home visit scheduling, and (where appropriate) clinical assessments enabled through video consultations. Furthermore, is the “Gold Line” service that provides video contact for people “approaching or in the last year of life” [47,48].

The Digital Health and Care Institute, based in Scotland and financially supported by the Scottish Government, reported that some 1.8 million (mainly older) people in the United Kingdom use such services, with some also benefiting from vital sign monitoring. Most of these services, it can be noted, have their origins in housing and social care services. The TSA (formerly the Telecare Services Association) meanwhile called for their service provider members to “engage with health and social care partners” to plan for the COVID-19 response and be ready “for increased demand from vulnerable service users” [49]. It follows that many telecare services in the United Kingdom have quickly reshaped their offerings to enable staff, normally located at their monitoring and control centers, to work from home; implement new practices for visiting staff (eg, to undertake assessments or respond to urgent circumstances); and adopt, where possible, self-installation procedures (where home “hubs” are delivered for simple connection to a telephone line or internet connection).

Telehealth service development in relation to COVID-19 in the United Kingdom appears, therefore, to be initially slow. The Scottish exception, similar to Australia, will build on experience that was driven by the needs of rural, remote, and island communities. Its program includes several recognized elements of telehealth such as “home and mobile health monitoring,” videoconferencing, and telecare. It also includes the use of a “bespoke” videoconferencing system for people at home (with internet-linked computers) or who are “on the move” (via smartphones). These services are not just concerned with health-related consultations but also links, at least for Scotland’s most rural health service, with dispensing practices to facilitate “better pharmaceutical management” [50,51].

For the United Kingdom overall, therefore, the COVID-19 outbreak was a major “jolt” to the National Health Service (NHS) that had been and remains, in part, reluctant to embrace telehealth. A recent harbinger of necessary change was offered, however, in the Topol Review [39] that called, in the context of technological changes, for dramatic improvements in the England’s health and social care infrastructure (including the workforce) and associated changes in culture. In the review, telemedicine (a subset of telehealth) topped the list of the most relevant and necessary technological advances, followed by smartphone apps and remote monitoring facilitated (in part) through sensors, including those embedded within wearable devices. Relevant also is NHSX, established in 2019 [52] as an NHS “spin-off,” that is intended to lead, for England, the “largest digital health and social care transformation programme in the world,” with foci that include the interoperability of systems and an intent to guide ways in which benefits for the NHS can be harnessed from “big data” analytics. Their “tech plan,” including attention to apps, was under development in 2020. NHSX is, according to the NHS England notice of March 17, 2020, leading work relating to telehealth developments in the primary care sector.

In response to the question as to why telehealth had not (to date) been developed further in the United Kingdom, Professor Trish Greenhalgh put it succinctly in a webinar on March 18, 2020 [40]. She affirmed, referring to clinicians, that they “*didn’t have any particular reason to use [telehealth]. They didn’t see a clinical need*,” adding that “*running a service with video-consultations as a main component involves major changes in workflows and also changes in professional interactions. And it feels a bit weird* [for them] *to be consulting either by telephone or video when you could just bring the patient in and look at them - as you were taught*.” Other work by Greenhalgh is relevant in exploring video consultations [53] and remote assessments [54].

Useful in this context is the release by the (UK) Royal College of General Practitioners of “top tips” for telephone consultations in the context of COVID-19 [55] and preliminary “video-consultation information” for GPs developed by the NHS in collaboration with the University of Oxford and drawing on guidance produced by the Scottish Government [56].

In summary, the *reach* of new telehealth initiatives to older people in the United Kingdom is uncertain. Many (older) people do not have smartphones or computers. Some, depending on location, will have poor (or no) connectivity. In the United Kingdom, furthermore, although there is good NHS *intent* to reach all those in need (as part of their universal service obligation), the rollout of telehealth services, despite the urgency of COVID-19, may be slow—though lessons will be learned from both Scotland and outside of the United Kingdom.

### United States of America

On March 17, 2020, the United States announced the “dramatic” expansion of telehealth services via tele- and videoconferencing, with people able to use these services over the ensuing 6 months through such platforms as Skype or Facetime and with the waiving of “other” normal requirements [57]. The waiving of regulations applied to the state authorized Medicare funded services that operate throughout the United States [58] and followed growing concerns about the COVID-19 outbreak. A further US $2 trillion (of which US $200 million was earmarked for telehealth) was promised through the Coronavirus Aid, Relief, and Economic Security Act that passed through the Senate on March 25, 2020, [59] and was signed into law on March 27.

Realization of the need for such urgent action was partly prompted by an article, published on March 11, 2020, by Hollander and Carr [24]. This affirmed that, in the context of COVID-19, “direct to consumer (or on-demand) telemedicine...is both patient-centered and conducive to self-quarantine, and it protects patients, clinicians and the community from exposure.” In place, furthermore, was an infrastructure in the United States that would, they envisaged, facilitate telehealth’s (telemedicine’s) greater use, with programs already in place for 50 of the country’s state health systems.

That initial week (commencing around March 11, 2020) was marked in the United States by an “explosion of demand” that “slammed into hospitals [that were] used to delivering telehealth consults for only a handful of patients a day.” Cleveland Clinic and Jefferson Health were reported as having fifteen- and twenty-fold increases, respectively, in telehealth visits in a week. In addition, Penn Medicine, because of their increased demand, “increased the number of practitioners delivering remote consults from six to 60” [25]. Further increases were expected. Additional impetus was added to the moves because of the applicability of the Imperial College London report that pointed to the equal (though far bigger in population terms) threat of deaths to the United States [45].

By March 17, 2020, the American Association of Retired Persons, the largest representative body of older people worldwide, had posted an item on their website that explained, in reassuring terms, what to expect in a virtual visit [60]. The author of the item affirmed that a virtual visit was “very similar to what would happen in person,” advising people to be ready to respond to questions, “to have a pen and paper handy,” and to be increasingly equipped to take physiological measures at home, notably blood pressure and temperature. The Centers for Disease Control and Prevention [61] had published interim guidance for community health staff that included the need to “explore alternatives to face-to-face triage and visits” and “identify staff to conduct telephonic and telehealth interactions,” with “protocols so that staff can triage and asses patients quickly.” The American Hospital Association [62] had, a year prior to the COVID-19 pandemic, called for “widespread elimination of geographical and setting locations requirements” and an expansion of “types of technology,” including remote monitoring, that could be used.

The new Medicare rules began retrospectively, starting on March 6, 2020. The telehealth services in question were required to consult in “real-time” (ie, asynchronous and store and forward communication was not included), and the prior restriction to beneficiaries in rural and remote areas was removed. Reimbursement rates for service providers were set at the same rate as for face-to-face visits. Importantly, all eligible Americans became able to link to telehealth services “through video chat and online patient portals” referred to as “virtual check-ins” [59].

The range of staff engaged by service providers (at “originating” sites) was widened, overall including doctors, nurse practitioners, licensed clinical social workers, and clinical psychologists, with ordinary consultations, as well as health screening and mental health counselling, being able to be undertaken. Health staff (including nurse practitioners and physician assistants, where necessary, at “distant” sites) became eligible for payment [63]. Although the focus was on Medicare, it can be noted that several of the private insurance service providers were starting to waive costs for remote assessments and consultations, reckoning on the health benefits that would ensue [64].

What superficially may look like a consistent approach in the United States (focused mainly on videoconferencing) hides variation between the funding and administrative frameworks that operate in different states. Dinesen et al [22] reported on a “fragmented” system in the United States where the “use of technologies can create jurisdictional conflicts, policy conflicts and remain tangential to care practices rather than integrated in [the] health care infrastructure.” In 2019, the Center for Connected Health Policy [65] affirmed that “no two states are alike in how telehealth is treated, despite some similarities in the language used.”

A useful fact sheet for telehealth was, however, provided by the American Hospital Association [62]. This noted that, in 2017, three-quarters of US hospitals connected “patients and consulting practitioners at a distance,” albeit that there were barriers to wider adoption. All states provided reimbursement under Medicaid “for some form of live video,” but less than half were reimbursed for “store and forward” [62]. There are many people, furthermore, who were recognized as “low-income or uninsured” and who “may have no choice but to pay out-of-pocket for these services” [66]. Only a minority of Americans, in fact, are on Medicare, with Cahan [67] arguing that “telehealth must also reach these 281 million individuals in the under-resourced nooks and crannies of the US.”

A US example of a telehealth service is provided by St Luke’s Health, which operates a “virtual care center” in Boise, Idaho. The center and its staff provide video consultations and remote patient monitoring for people at other linked health care facilities, in their homes, or (via mHealth) on the move. Importantly, the service approach is seen as evolving from one that is more reactive and responsive to health needs as they arise to one that is proactive in supporting people’s day to day health [68].

Finally, mention must be made of the fact that the United States has well developed personal response services (social alarms) that are frequently in place to support patients after hospital discharge or are otherwise used to enable people to contact responders after a fall or in other necessitous circumstances. Most of these are private sector services and some are seeking to evolve as telehealth services. They echo in their form the social alarm services in Australia and the United Kingdom.

As for all three countries, the *reach* of telehealth initiatives to older people in the United States, despite the urgency around COVID-19, is uncertain. Many people do not have smartphones or computers. Some people, depending on their location, will have poor (or no) connectivity. In the United States there are, furthermore, issues around the cost of services where (older) people are not eligible for Medicare.

## Discussion

This paper has exposed differences in the way that Australia, the United Kingdom, and the United States responded, in the 2-week period beginning March 12, 2020, to the promotion and use of telehealth to combat the COVID-19 pandemic. These differences relate to their prior experiences with telehealth, the different health, and to some extent, social care, contexts, and cultures; the extent that specific attention was given to older people; the respective geographies (most notably in relation to rural areas); and the linked funding frameworks. Differences in understandings of definitions or the breadth of telehealth were also indicated, reflecting the nature of extant services within the countries in question.

Regarding the health context, a strong link to long-standing universal welfare models is signaled for both Australia and the United Kingdom. In the United Kingdom, a major part of telehealth and related service provision is via public and third sector (charitable or nonprofit) organizations. In Australia, provision is both from these and the private sector. The welfare element is, however, also there for the United States where Medicare and Medicare programs seek to ensure that the needs of at least some of those who are most vulnerable are met, albeit with most people normally accessing services through private providers.

All three countries give attention to older people. An interesting aspect of this, especially in Australia and the United Kingdom, are the moves toward cultural change within services, being reflected in an increased understanding of the potential of technologies to empower their users. The use of apps (notable in the United Kingdom) and voice assistant devices in the home are pointers to this, with indications in all three countries of some moves, within the context of telehealth, toward encouraging greater self-management.

The different geographies of the three countries have been observed as influential. This, in part, reflects the fact that some (often pioneer) telehealth services, were born of necessity—arising from rurality, remoteness, and island locations, and workforce shortages [69]. This is particularly the case for Australia. It is interesting to note, therefore, the actions by governments in both Australia and the United States that waived restrictions on financial support for service provision in nonrural areas. In addition, regardless of the future of telehealth, there is no doubt as to its real achieved benefits as perceived by (older) people living in rural and remote regions.

Understandings of the meaning of telehealth in the three countries were clearly influenced by the nature of services that had been established, this then being consolidated by what was determined as eligible for funding. Hence the US “model” based around video consultations can be contrasted with the less rigidly framed, but arguably more inclusive, services in Australia and the United Kingdom. The US model may, nevertheless, prove to be a useful foundation for extended services that are able to respond to the COVID-19 pandemic. By contrast, the rapid building or strengthening of telehealth services in Australia and the United Kingdom as a response to COVID-19 could prove more problematic, in the sense that greater attention will be necessary to establishing or putting in place adjustments to staffing and operational procedures able to both ensure reasonable quality standards (for technologies and services) and to respond to the certain increase in demand.

As noted in STAT [25], for the United States, “the administrative challenges are numerous. They include training doctors to deliver virtual care...there are also technology set up challenges for new users as well as a shortage of bandwidth as the internet groans under the strain of increasing use.” All three countries, meanwhile, could consider the extent to which “nurse-practitioners” and “physician assistants” could play a greater part together with social care staff (and assisted by the technologies) in the operation of telehealth services. There are, in addition, all kinds of related challenges for information governance and the inevitably time-consuming tasks associated with quality testing of both the technologies and the related services. In addition, even though funding may be provided in this extraordinary time, the questions remain as to whether this will prove adequate to support the running of viable telehealth businesses in the COVID-19 context.

Common to all three countries is their commitment to at least basic service quality safeguards. These are essential and will need to be built upon if telehealth is to take its place within established health services. The COVID-19 pandemic is forcing the changes, and the question of service sustainability will, therefore, remain. We are some way from what Cahan [67] referred to as telehealth becoming “finally mainstream – overnight(ish),” and we are some way away from loosening the ties that bind us to “the delivery of services in bricks-and-mortar campuses and clinics” and face-to-face consultations and care, “where infection transmission is too easily facilitated” [25].

What begins to come through, however, is a sense of an increasing and shared recognition of how the technologies that we associate with telehealth are not only vehicles for the provision of services but also offer a means for people’s empowerment. This empowerment applies to all, including older people for whom there is an imperative for equal effort to be expended in ensuring both the form of the technologies and services is appropriate to facilitate their use. Although with telehealth, the extent of personal contact in the form of *presence* and *touch* has been noted as currently out of reach; tele- or video consultations may offer a route toward helping address the twin scourges of loneliness and social isolation that can afflict some older people. Linked with this is the ability, in the context of COVID-19, of telehealth to make the links that give comfort to (older) people when they are ill, self-isolating, or being confined. In addition, for (older) people who are more mobile, there is the often-cited telehealth benefits of not needing to travel to health facilities, with the concomitant inconvenience and cost to themselves and, frequently, accompanying persons.

Further than this, the empowerment of older people living with mild dementia and their caregivers during COVID-19 has been demonstrated in Spain through television-based health and social support interventions, and the provision of telephone-based support [70]. Telehealth in the form of television-based support went, therefore, beyond an initial objective of home support and is concerned with promoting “active aging” at home. The program has enabled caregivers to provide remote support and improved cognitive function with online memory exercises.

In summary, the picture that emerges is one of uncertainties and differences for the three countries but with an increasing awareness of the part that can, and probably must, be played by telehealth in the context of COVID-19. That part will potentially have a great benefit for older people who, it has been noted, are disproportionately impacted by the virus.

However telehealth services develop in this context, there is the reassurance, of a shared concern in the three countries for at least basic quality standards to be maintained. To do this, and whether or not the immediate impact on telehealth services is sustained after the pandemic, there will be a commensurate need for telehealth (or, rather, the broader realm of digital health) to become integrated within health and social care service frameworks. In other words, telehealth must not be seen as an “alternative” form of health care. It follows that telehealth, regardless of the impact of COVID-19, must also become integrated within the training curricula for both health and social care professionals and practitioners.

## Abbreviations

ACRRM: Australian College of Rural and Remote Medicine
ACIITC: Aged Care Industry IT Council
AI: artificial intelligence
CFR: case-fatality rate
COVID-19: coronavirus disease
GP: general practitioner
mHealth: mobile health
NHS: National Health Service
RACGP: Royal Australian College of General Practitioners
WSD: Whole System Demonstrator
